# Modulation of macrophage activation by prostaglandins

**DOI:** 10.1155/S0962935196000026

**Published:** 1996-02

**Authors:** L. Sautebin, R. Carnuccio, F. D'Acquisto, M. Di Rosa

**Affiliations:** 1 Department of Experimental Pharmacology University of Naples “Federico II” Via Montesano 49 Naples 80131 Italy

## Abstract

The effect of prostaglandtn E_2_, iloprost and cAMP on both nitric oxide and tumour necrosis factor-α release in J774 macrophages has been studied. Both prostaglandin E_2_ and iloprost inhibited, in a concentration-dependent fashion, the lipopolysaccharide-induced generation of nitric oxide and tumour necrosis factor-α. The inhibitory effect of these prostanoids seems to be mediated by an increase of the second messenger cAMP since it was mimicked by dibutyryl cAMP and potentiated by the selective type IV phosphodiesterase inhibitor RO-20-1724. Our results suggest that the inhibition of nitric oxide release by prostaglandin E_2_ and iloprost in lipopolysaccharide-activated J774 macrophages may be secondary to the inhibition of tumour necrosis factor-α generation, which in turn is likely to be mediated by cAMP.


Mediators of Inflammation 5, 14–17 (1996)

### Modulation of macrophage activation by prostaglandins

L. Sautebin,[CA] R. Carnuccio, F. D'Acquisto and M. Di Rosa

Department of Experimental Pharmacology, University of Naples "Federico II", Via Montesano 49, 80131 Naples, Italy.
Fax: (+39) 81 7486403.

[CA]Corresponding Author

**THE effect of prostaglandin $E_2$, iloprost and cAMP on both nitric oxide and tumour necrosis factor-α release in J774 macrophages has been studied. Both prostaglandin $E_2$ and iloprost inhibited, in a concentration-dependent fashion, the lipopolysaccharide-induced generation of nitric oxide and tumour necrosis factor-α. The inhibitory effect of these prostanoids seems to be mediated by an increase of the second messenger cAMP since it was mimicked by dibutyryl cAMP and potentiated by the selective type IV phosphodiesterase inhibitor RO-20-1724. Our results suggest that the inhibition of nitric oxide release by prostaglandin $E_2$ and iloprost in lipopolysaccharide-activated J774 macrophages may be secondary to the inhibition of tumour necrosis factor-α generation, which in turn is likely to be mediated by cAMP.**

**Key words:** cAMP, Macrophages, Nitric oxide, Phosphodiesterase inhibitor, Prostaglandins, Tumour necrosis factor

### Introduction

Macrophages activated with bacterial lipopolysaccharide release a variety of mediators including nitric oxide (NO), tumour necrosis factor (TNF-α) and prostaglandins, namely prostaglandin $E_2$ ($PGE_2$) and prostacyclin ($PGI_2$).[1–3] The cytotoxic properties of activated macrophages depend, at least in part, on the biological activities of these mediators. Thus the synthesis of NO from the amino acid L-arginine has been shown to be a major cytotoxic mechanism of activated macrophages.[1] Moreover NO has been identified as an effector molecule of the cytotoxic effects produced by TNF-α in bovine endothelial cells.[4] Conversely, $PGE_2$ and $PGI_2$ inhibit the cytotoxic properties of activated macrophages.[3] Furthermore, increasing evidence suggests that the interaction existing within the biological actions of NO, prostaglandins and TNF-α may result in a mutual regulation of their synthesis and/or release. In fact TNF-α has been reported to induce NO synthase in murine peritoneal macrophages[5] and bovine endothelial cells.[4] TNF-α increases $PGE_2$ production in human synovial cells and human dermal fibroblasts,[6] mouse peritoneal macrophages[7] and rat Kupffer cells,[8] as well as $PGI_2$ generation in human endothelial cells.[9] Conversely, $PGE_2$ has been shown to down-regulate the generation of TNF-α in macrophages of different origin[2,10] and HL-60 cells.[11] Moreover it has been reported that the release of TNF-α by macrophages is inhibited by cAMP, a second messenger for both $PGE_2$ and $PGI_2$.[10–12]

We have shown that $PGE_2$ and the stable analogue of $PGI_2$, iloprost, inhibited the induction of NO synthase in lipopolysaccharide-activated J774 murine macrophages.[13] We hypothesized that the inhibition of NO synthase induction could be secondary to an increase in cAMP levels in the activated macrophages. This hypothesis was also supported by the inability of both prostaglandin $F_{2\alpha}$ ($PGF_{2\alpha}$) and the stable analogue of thromboxane $A_2$ ($TXA_2$), U46619, which do not enhance cAMP levels, to modify NO generation. However, since TNF-α induces No synthase in murine macrophages[7] and $PGE_2$ has been shown to inhibit TNF-α release from these cells,[2] the inhibition of NO synthase induction by $PGE_2$ could also be secondary to the down-regulation of TNF-α in J774 cells.

In the light of the above considerations we have studied the effect of $PGE_2$, $PGI_2$ and cAMP on the production of NO and TNF-α by lipopolysaccharide-stimulated J774 macrophages.

### Materials and Methods

The murine monocyte macrophage cell line J774 (American Tissue Culture Catalogue TIB 67, page 231) was grown in Dulbecco's modified Eagle's medium (Gibco) at 37°C as described previously.[13] The cells were plated in 24-well culture plates (Falcon) at a density of $2.5 \times 10^5$ cells/ml and allowed to adhere at 37°C in 5% $CO_2$/95% air for 2 h. Thereafter the medium was replaced with fresh medium, cells were activated with lipopolysaccharide (0.1 μg/ml) from *Salmo-*

© 1996 Rapid Science Publishers

*nella thyphosa* (Difco) and incubated in the presence of various concentrations (see Results) of test compounds: $PGE_2$, $PGF_{2\alpha}$ and dibutyryl cAMP (Sigma), iloprost (Schering), RO-201724 (Biomol).

NO was measured as nitrites ($NO_2^-$, nmol per $10^6$ cells) accumulated in the incubation media 24 h after lipopolysaccharide challenge. A spectrophotometric assay based on the Griess reaction was used.[14]

The level of TNF-α (U/ml) in the cell medium, 3 h after lipopolysaccharide challenge, was assessed in WEHI-164 cells by a biological assay using recombinant human TNF-α (Sigma) as reference standard and rabbit antimurine TNF-α (Genzyme) antiserum which cross-reacts with rat TNF-α in order to assess the specificity of TNF-α-dependent cytolytic activity.[15]

Data are expressed as percent of control release (mean ± standard error of the mean of *n* observations). Comparisons were made by the unpaired two-tailed Student's *t*-test. The level of statistically significant difference was defined as $p < 0.05$.

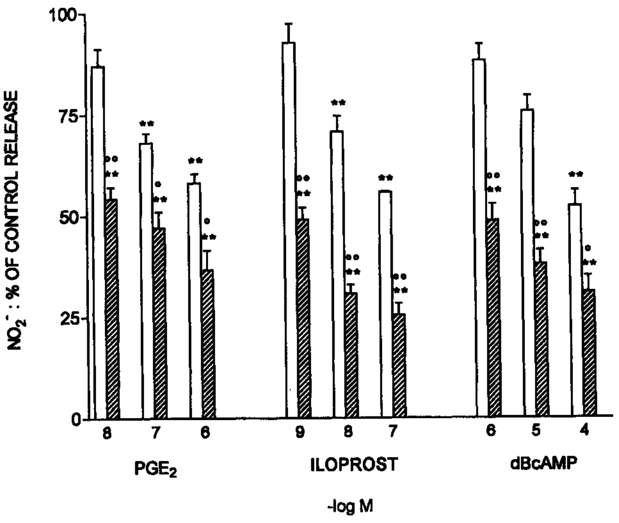

FIG. 1. Effect of prostaglandin $E_2$ ($PGE_2$), iloprost and dibutyryl cAMP (dBcAMP) on $NO_2^-$ generation by J774 murine macrophages 24 h after lipopolysaccharide challenge (0.1 μg/ml) in the absence (open bars) or presence (hatched bars) of RO-201724 $10^{-4}$ M. Data are expressed as mean ± standard error of the mean of five to six experiments. **$p < 0.01$ *vs.* control (lipopolysaccharide alone = 100% release); °$p < 0.05$, °°$p < 0.01$ *vs.* the corresponding concentration of the inhibitor in the absence of RO-201724.

### Results

The production of $NO_2^-$ by unstimulated J774 macrophages was undetectable (< 1 nmol per $10^6$ cells in 24 h, $n = 6$). The cells stimulated with lipopolysaccharide (0.1 μg/ml) released, as previously reported (Marotta *et al.*[13]), substantial amounts of NO, measured as $NO_2^-$ (64.5 ± 2.3 nmol per $10^6$ cells in 24 h, $n = 8$). When the cells were stimulated in the presence of dibutyryl cAMP ($10^{-4}$ – $10^{-6}$ M), $PGE_2$ ($10^{-6}$ – $10^{-8}$ M) or iloprost ($10^{-7}$ – $10^{-9}$ M), added concomitantly with LPS, a concentration-dependent decrease of $NO_2^-$ accumulation was observed (Fig. 1). $PGE_2$, iloprost and cAMP, at any of the concentrations tested, did not affect $NO_2^-$ generation when added to the cells 6 h after lipopolysaccharide challenge (data not shown). Conversely, $PGF_{2\alpha}$ ($10^{-6}$ – $10^{-8}$ M) did not significantly affect $NO_2^-$ accumulation when added concomitantly with lipopolysaccharide or 6 h later (data not shown). The inhibition produced by $10^{-4}$ M dibutyryl cAMP was about 50% ($p < 0.01$) and was virtually superimposable on the inhibition produced by $10^{-6}$ M $PGE_2$ and $10^{-7}$ M iloprost. The selective inhibitor of cAMP-specific phosphodiesterase type IV, RO-201724,[16] significantly ($p < 0.01$) increased the inhibition induced by dibutyryl cAMP. In fact, in the presence of $10^{-4}$ M RO-201724, the inhibition of $NO_2^-$ accumulation by $10^{-4}$ M dibutyryl cAMP was significantly increased from about 50% to about 70% (Fig. 1). RO-201724 also potentiated the inhibitory action of $PGE_2$ and iloprost on $NO_2^-$ generation (Fig. 1). RO-201724 alone ($10^{-4}$ M) did not produce any effect (data not shown).

We have also studied the effect of $PGE_2$, $PGI_2$ (iloprost) and cAMP (dibutyryl cAMP) on TNF-α release from lipopolysaccharide-activated J774 cells. Three h after lipopolysaccharide-challenge these cells released 297 ± 20.5 U/ml of TNF-α ($n = 8$), compared to the undetectable release by unstimulated cells (< 1 U/ml in 3 h, $n = 8$). Both $PGE_2$ ($10^{-6}$ – $10^{-8}$ M) and iloprost ($10^{-7}$ – $10^{-12}$ M) produced a concentration-dependent inhibition of TNF-α release (Fig. 2). $PGF_{2\alpha}$ ($10^{-6}$ – $10^{-8}$ M) did not produce any effect (data not shown). It is interesting to note that iloprost, as observed for $NO_2^-$ generation, was more potent than $PGE_2$, also an inhibitor of TNF-α release. Moreover, both prostanoids significantly inhibited ($p < 0.01$) TNF-α release at concentrations which were virtually ineffective on $NO_2^-$ production ($10^{-8}$ M $PGE_2$ or $10^{-9}$ M iloprost). The phosphodiesterase inhibitor RO-201724 ($10^{-4}$ M) potentiated the effect of iloprost for significant inhibition ($p < 0.01$) of TNF-α release was observed at concentrations of $10^{-11}$ and $10^{-12}$ M, (10.5 ± 1.1% and 41.1 ± 3.7% of control release, respectively; $n = 4$). Dibutyryl cAMP ($10^{-4}$ – $10^{-6}$ M) inhibited lipopolysaccharide-induced TNF-α release in a concentration-dependent fashion (Fig. 2). Moreover, as observed for $PGE_2$ and iloprost, dibutyryl cAMP was more potent as an inhibitor of TNF-α release

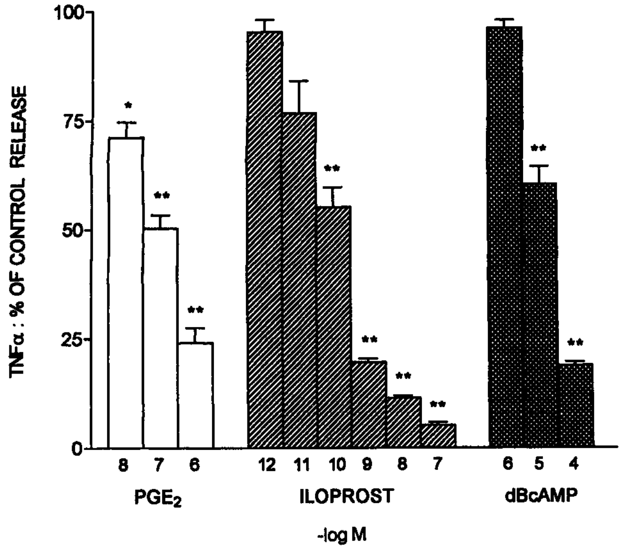

FIG. 2. Effect of prostaglandin $E_2$ ($PGE_2$, open bars) iloprost (hatched bars) and dibutyryl cAMP (dBcAMP, cross-hatched bars) on TNF-α release by J774 murine macrophages 3 h after lipopolysaccharide challenge (0.1 μg/ml). Data are expressed as mean ± standard error of the mean of five to six experiments. *$p < 0.05$, **$p < 0.01$ *vs.* control (lipopolysaccharide alone = 100% release).

than as an inhibitor of $NO_2^-$ generation, since at a concentration of $10^{-5}$ M, which was poorly effective for $NO_2^-$ accumulation, a significant inhibition ($p < 0.01$) of TNF-α release was produced.

### Discussion

We have shown[13] that $PGE_2$ and $PGI_2$, and its stable analogue iloprost, which are known to activate adenylate cyclase, inhibit the induction of NO synthase in lipopolysaccharide-activated J774 macrophages. We also demonstrated that this action was not shared by $PGF_{2\alpha}$ and the stable analogue of $TXA_2$, U46619, which do not enhance cAMP levels. The results of the present study show that cAMP, as its permeable form dibutytryl cAMP, inhibits lipopolysaccharide-induced NO release by J774 macrophages, and confirm that this release is inhibited by $PGE_2$ and iloprost but not by $PGF_{2\alpha}$. The selective type IV phosphodiesterase inhibitor, RO-201724,[16] potentiated not only the inhibitory effect of cAMP but also the effect of the two prostanoids. In the light of these findings our results strongly suggest that the inhibitory action of the two prostanoids on NO synthase induction in lipopolysaccharide-activated J774 macrophages is mediated by cAMP. This evidence is also supported by recent results showing that in murine peritoneal macrophages, cAMP is an intermediate in the down-regulation of NO synthase by prostaglandins.[17]

Our results also show that $PGE_2$ and iloprost, but not $PGF_{2\alpha}$, inhibit in a concentration-dependent fashion of lipopolysaccharide-induced TNF-α production by J774 macrophages. This effect was exhibited also by cAMP. The phosphodiesterase inhibitor RO-201724 potentiated this action. $PGF_{2\alpha}$ was unable to inhibit lipopolysaccharide-induced TNF-α release by J774 macrophages. These data suggest that, as observed for NO generation, the inhibitory action of the two prostanoids on TNF-α release is likely to be mediated by cAMP. In this respect it is interesting to note that all the tested compounds were more potent in inhibiting TNF-α than $NO_2^-$ production. Since NO seems to be the effector molecule of the cytotoxic activity of TNF-α,[5] and $PGE_2$ and iloprost modulate the production of TNF-α and NO,[17] it could be hypothesized that the inhibitory action of the two prostanoids on NO synthase induction might be secondary to the inhibition of TNF-α generation which seems to be mediated by cAMP. This hypothesis appears conceivable considering that in macrophages lipopolysaccharide stimulates not only TNF-α and NO but also a sustained production of the prostaglandins, following the expression of inducible cyclooxygenase.[18] In this light the down-regulation of TNF-α and consequently of NO production by endogenous prostaglandins may represent a relevant feed-back mechanism in modulating the cytotoxic effects of a sustained NO production following immunological stimulation.

### References

1. Hibbs Jr JB, Taintor RR, Vavrin Z, Rachlin EM. Nitric oxide: a cytotoxic activated macrophages effector molecule. *Biochem Biophys Res Commun* 1988; **157**: 87–94.
2. Kunkel S, Spengler M, May MA, Spengler M, Larick J, Remick D. Prostaglandin $E_2$ regulates macrophages-derived tumor necrosis factor gene expression. *J Biol Chem* 1988; **263**: 5380–5384.
3. Bonta IL, Ben-Efraim S. Leukotrienes and prostaglandins mutually govern the antitumoral potential of macrophages. In: Garaci E, Paoletti R, Santoro MG, eds. *Prostaglandins and Cancer Research.* Heidelberg: Springer Verlag, 1987; 193–201.
4. Estrada C, Gomez C, Martin S, Moncada S, Gonzalès C. Nitric oxide mediates tumor necrosis factor-α cytotoxicity in endothelial cells. *Biochem Biophys Res Commun* 1992; **186**: 475–482.
5. Drapier JC, Wietzerbin J, Hibbs Jr JB. Interferon-γ and tumor necrosis factor induce the L-arginine-dependent cytotoxic effector mechanism in murine macrophages. *Eur J Immunol* 1988; **18**: 1587–1592.
6. Dayer J, Beutler B, Cerami A. Cachectin/tumor necrosis factor stimulates collagenase and prostaglandins $E_2$ production by human synovial cells and dermal fibroblasts *J Exp med* 1985; **162**: 2163–2167.
7. Backwich P, Chensue S, Larrick J, Kunkel S. Tumor necrosis factor stimulates interleukin-1 and prostaglandin $E_2$ production in resting macrophages. *Biochem Biophys Res Commun* 1986; **136**: 94–101.
8. Peters T, Karck U, Decker K. Interdependence of tumor necrosis factor, prostaglandin $E_2$ and protein synthesis in lipopolysaccharide-exposed rat Kupffer cells. *Eur J Biochem* 1990; **191**: 583–589.
9. Kawakami M, Ishibashi S, Ogawa H, Murase T, Takaku F, Shibata S. Cachectin/TNF as well as interleukin-1 induces prostacyclin synthesis in cultured vascular endothelial cells. *Biochem Biophys Res Commun* 1986; **141**: 482–487.
10. Renz H, Gong JH, Schmidt A, Nain M, Gemsa D. Release of tumor necrosis factor-α from macrophages. Enhancement and suppression are dosedependently regulated by prostaglandins. *J Immunol* 1988; **141**: 2388–2393.

11. Mohri M, Spriggs DR, Kufe D. Effects of lipopolysaccharide on phospholipase $A_2$ activity and tumor necrosis factor expression in HL-60 cells. *J Immunol* 1990; **144**: 2682–2687.
12. Endres S, Fulle HJ, Sinha D, Stoll D, Dinarello CA, Gerzer R, Weber PC. Cyclic nucleotides differentially regulate the synthesis of tumour necrosis factor-α and interleukin-1β by human mononuclear cells. *Immunology* 1991; **72**: 56–60.
13. Marotta P, Sautebin L, Di Rosa M. Modulation of the induction of nitric oxide synthase by eicosanoids in the murine macrophages cell line J774. *Br J Pharmacol* 1992; **107**: 640–641.
14. Green LC, Wagner DA, Glogowsky JS, Skipper PL, Wishnok JS, Tannenbaum SR. Analysis of nitrate, nitrite and [15-N] nitrite in biological fluid. *Analyt Biochem* 1982; **126**: 131–136.
15. Garrelds IM, Zijlstra FJ, Tak CJAM, Bonta IL, Beckman I, Ben-Efraim S. A comparison between two methods for measuring necrosis factor in biological fluids. *Agents Actions* 1993; **38**: C90–91.
16. Reeves ML, Leigh BK, England PJ. The identification of a new cyclic nucleotide phosphodiesterase activity in human and guinea-pig cardiac ventricle. *Biochem J* 1987; **241**: 535–541.
17. Raddassi K, Petit SF, Lemaire G. LPS-induced activation of primed murine macrophages is modulated by prostaglandins and cyclic nucleotides. *Cellular Immunology* 1993; **149**: 50–64.
18. Masferrer JL, Seibert K, Zweifel B, Needleman P. Endogenous glucocorticoids regulate an inducible cyclooxygenase enzyme. *Proc Natl Acad Sci USA* 1992; **89**: 3917–3921.

**Received 11 October 1995; accepted 8 November 1995**
